# Nanocrystalline diamond surfaces for adhesion and growth of primary neurons, conflicting results and rational explanation

**DOI:** 10.3389/fneng.2014.00017

**Published:** 2014-06-11

**Authors:** Silviya M. Ojovan, Mathew McDonald, Noha Rabieh, Nava Shmuel, Hadas Erez, Milos Nesladek, Micha E. Spira

**Affiliations:** ^1^Department of Neurobiology, The Alexander Silberman Institute of Life Science, The Hebrew University of JerusalemJerusalem, Israel; ^2^Institute for Materials Research in MicroElectronics – Interuniversity Micro Electronics Centre, Hasselt UniversityAntwerp, Belgium; ^3^The Harvy M. Kruger Family Center for Nanoscience, The Hebrew University of JerusalemJerusalem, Israel

**Keywords:** nanocrystalline diamonds, cultured neurons, poly-d-lysine, cell adhesion, calcium imaging, network connectivity

## Abstract

Using a variety of proliferating cell types, it was shown that the surface of nanocrystalline diamond (NCD) provides a permissive substrate for cell adhesion and development without the need of complex chemical functionalization prior to cell seeding. In an extensive series of experiments we found that, unlike proliferating cells, post-mitotic primary neurons do not adhere to bare NCD surfaces when cultured in defined medium. These observations raise questions on the potential use of bare NCD as an interfacing layer for neuronal devices. Nevertheless, we also found that classical chemical functionalization methods render the “hostile” bare NCD surfaces with adhesive properties that match those of classically functionalized substrates used extensively in biomedical research and applications. Based on the results, we propose a mechanism that accounts for the conflicting results; which on one hand claim that un-functionalized NCD provides a permissive substrate for cell adhesion and growth, while other reports demonstrate the opposite.

## INTRODUCTION

The construction of efficient brain–machine interfaces (BMI) relies, to a large extent, on the use of biocompatible materials that can withstand the harsh biological solutions comprising the environment in which living cells operate. A promising substrate for such BMIs is the family of nanocrystalline diamond (NCD) which is a continuous layer of nanoscopic diamond crystals embedded in a nanoscale matrix of sp2 and disordered carbon (1-10%; Daenen et al., 2009). NCD exhibits chemical and biochemical inertness, high corrosion resistance, excellent mechano-optical properties and large surface area (Fendrych et al., 2010). While intrinsic NCD is electrically insulating, it can be doped with boron to form semiconducting or even metallically conducting films with tunable surface chemistry (Singh et al., 2010; Zivcova et al., 2013). These properties make NCD and Boron-Doped NCD (BNCD) a very attractive material for electrodes or electrode coatings for the development of implantable electrodes for the restoration of sensory functions such as in cochlear or retinal implants (Xiao et al., 2006; Hadjinicolaou et al., 2012; Ganesan et al., 2014), to electrically communicate between neurons or muscles and peripheral prosthesis (Ariano et al., 2009) and for deep brain stimulation and recordings.

A major concern for using NCD coatings for multi-electrode arrays (MEAs) is whether it can serve as a permissive surface for post mitotic neuron adhesion and regenerative regrowth.

The general impression from the literature is that NCD substrates have superior biocompatible and adhesion permissive properties. Thus, it was reported that proliferating cells, including fibroblast, epithelial cells, and various cell lines, adhere and develop on bare (chemically non-functionalized) NCD surfaces. For example, Amaral et al. (2009) demonstrated that fibroblast cell line L929 (a mouse permanent cell line) and human gingival fibroblast adhere and proliferate on NCD surfaces without hydrophilic treatment or surface functionalization. Klauser et al. (2010), revealed that epithelial cells attach within 24–72 h onto oxygen-terminated NCD substrates without further surface functionalization and without the addition of fetal bovine serum (FBS) to the growth medium. Using the GT1-7 proliferating cell line, which was used to represent neurons, Ariano et al. (2009) documented that these cells adhere to H- or O-terminated NCD substrates without surface functionalization. In an apparent consistency with the above observations, Thalhammer et al. (2010) reported that chemically un-functionalized monolayers of diamond nanoparticles, prepared by the minute detonation synthesis (the so called detonation nanodiamond-DND; Mochalin et al., 2012), promote adhesion and growth of cultured post mitotic hippocampal neurons; emphasizing that chemical surface functionalization does not improve the culture. This conclusion was recently supported also by Edgington et al. (2013). In the context of the potential use of NCD substrates for bidirectional electrical coupling of neurons or muscles with microelectrodes, it is important to note that beside the mechanical, chemical, and biological compatibilities of a given interface, the interfacing material for BMIs should be also electrically tunable. Although both materials (NCD and DND) are of the same chemical composition, e.g., carbon in sp3 bonding configuration with sp2 inclusions at the grain (NCD) or particle (DND) boundary (Ferrari and Robertson, 2004; Daenen et al., 2009) they differ in the term of technological capabilities. DND do not integrate into a compact layer; they cannot be used to construct flat or three-dimensional electrically conducting tracks. DND could be mainly used in combination with BNCD as a neuron adhesion promoting coating, however, DND can alter surface chemical properties of BNCD, by augmenting the Faradic currents. For example, it has been demonstrated by Holt et al. (2008) that DND particles on gold substrate surface lead to enhancement of redox reactions that can influence cellular processes by promoting the reduction of oxygen with potential production of harmful reactive oxygen species.

Driven by the potential use of NCD for fabrication of *in vitro* and *in vivo* bio-electronic platforms, we undertook here the examination of NCD surfaces (rather than DND particles) as substrates for post-mitotic primary vertebrate neurons. In contrast to the expectations based on the literature, we found that it is practically impossible to culture primary neurons, in a defined culture medium, on hydrogen or oxygen terminated NCD. Nevertheless, in view of the physical and chemical advantages of NCD for *in vitro* and *in vivo* applications, we have investigated whether chemical functionalization of NCD surfaces can confer permissive substrate properties for adhesion and growth of post mitotic neurons. We report that the problem of bare NCD to promote adhesion and growth of cultured primary neurons can be overcome using conventional poly-D-lysine (PDL)-laminin functionalization. Based on the results, we propose a mechanism that could account for the conflicting results, which on the one hand claim that un-functionalized NCD provides a permissive substrate for adhesion and growth of cells, while other reports demonstrate the opposite.

## MATERIALS AND METHODS

### NCD FABRICATION

The diamond films were grown using an Astex AX6550 microwave plasma enhanced chemical vapor deposition system (MW-PECVD) working at H_2_ (99%)/CH_4_ (1%) gas mixture and substrate temperature of 700°C. The substrate was cleaned using standard RCA1 and RCA2 (Radio Corporation America, Kern, 1990) wafer cleaning techniques and subsequently seeded by detonation nanodiamond (DND, 7 nm size) by spin-coating from an aqueous DND mono-dispersion (NanoAmando®B, NanoCarbo Research Institute, Japan) prior to the deposition. Hydrogen terminated NCD films were achieved by allowing pure hydrogen plasma in the system for 30 min after stopping the CH_4_ flow and subsequently cooling in hydrogen. UV ozone treatment for 30 min (Novascan, PSD digital Ozone system) was used on hydrogen terminated films to give NCD films anoxygen terminated surface. Surface termination was confirmed by XPS and the contact angle measurement, showing hydrophobic surfaces for H-terminated films with the contact angle of 15° and of 90–92° for O-terminated NCD films.

### SURFACE FUNCTIONALIZATION

Glass or NCD chips were washed and sterilized by incubation in ethanol 75% for 2 h. Thereafter the ethanol was thoroughly rinsed with double distilled water and then functionalized by 0.1 mg\ml PDL (Sigma–Aldrich) and 25 μg\ml laminin (Sigma–Aldrich) in sodium borate solution for 12 h prior to cells seeding.

### CELL CULTURE

Rat hippocampal neurons were obtained either from one day old new born rats or from 17 days old embryos, as described by Kaech and Banker (2006). Briefly, for embryonic hippocampal cultures the pregnant female was anesthetized, the embryos removed and decapitated. The embryonic or new born hippocampus were removed and treated with papain for 45 min (Sigma–Aldrich), and serially triturated. Cell density at plating was 250,000–500,000 cells/ml. Cells were seeded in attachment\seeding medium [Neurobasal medium, 5% FBS, 2% B27, 1%GlutaMAX (All from Life technologies), 1% Penicillin-Streptomycin Amphotericin B Solution (Biological Industries)]. 24 h (1 DIV) after culturing the seeding medium was replaced with serum-free maintenance\feeding medium (Neurobasal medium, 2% B27, 1% GlutaMAX, 1% Penicillin-Streptomycin Amphotericin B Solution). At 3 DIV 5 μM ara-c (Sigma–Aldrich) was added to prevent glial cell’s proliferation. Half of the maintenance medium was replaced every 3–5 days by astroglial conditioned medium. Hippocampal cultured cells were kept at 37°C in a humidified atmosphere of 5% CO_2_. Cultures were kept till 7–21 DIV. All procedures were approved by the Committee for Animal Experimentation at the Institute of Life Sciences of the Hebrew University of Jerusalem.

### IMMUNOHISTOCHEMISTRY

Cultured hippocampal cells were immunolabeled as previously described (Fendyur et al., 2011). Briefly, samples were fixed by 4% paraformaldehyde (Sigma–Aldrich) in Hank’s Balanced Salt Solution (HBSS, Biological Industries) for 30 min, washed with HBSS before membrane permeabilization with 0.1% TritonX-100 (BDH Chemicals) in HBSS for 30 min. After washes with Tween 0.1% (J.T.Baker) in HBSS, cells were incubated for 1 h in blocking solution [BS, 2% chicken albumin (Sigma–Aldrich) in Tween 0.1%]. Then samples were incubated with primary antibodies in 1% BS, overnight at 4°C: neurons were labeled for neuron-specific intermediate filaments with mouse anti neurofilament antibodies; Glial cells were labeled for glial fibrillary acidic protein (GFAP) with primary anti-GFAP rabbit monoclonal antibodies. The next day the samples were washed repeatedly with 0.1% Tween and incubated with secondary antibodies in 1% BS for 1 h: goat anti-mouse secondary antibodies conjugated to Cy2 (Jackson ImmunoResearch Laboratories, Inc), and goat anti-rabbit secondary antibodies conjugated to Cy3 (Life technology). Cells were counterstained with the nuclear marker DAPI (Sigma–Aldrich) for 1 h, at room temperature. Samples were washed with HBSS, and stored at 4°C in anti-fade n-propyl gallate (Sigma–Aldrich) solution in 50% glycerol till imaging. Confocal imaging of the immunolabeled cultures was done using D-Eclipse C1 imaging system (Nikon) mounted on an Eclipse TE-2000 microscope (Nikon). Images were collected and processed using EZ-C1 software (Nikon). Scanning was done in sequential mode: red was excited with 543 nm He–Ne laser and collected with 605 ± 75 band pass filter; green was excited with 488 nm Argon laser and collected with 515 ± 30 band pass filter; blue excited with 405 nm Diode and collected with 450 ± 35 band pass filter. Images were prepared using the open-source image analysis program ImageJ (NIH, USA) and Photoshop CS6.

### CALCIUM IMAGING AND FORMATION OF A CONNECTIVITY MAP

Ten to 14-day-old cultures were incubated for 45 min in a 37°C incubator in a 5 μM fluo-4 (AM; Molecular Probes) dissolved in HBSS. The fluo-4 AM solution was washed away with HBSS and the culture was then incubated in fluo-4 free medium for 15 min. Cells were imaged with a 20 × objective (NA = 0.75) using the confocal microscope system described above. Seconds long stimulation of a single neuron at the center of the field of view was applied with a fire polished patch electrode and the response imaged by the frame-scan modes. The second long stimulation led to a buildup of intracellular calcium levels that could be detected by the low NA objective and the slow laser scan used (3.9 s/frame). Sequential confocal fluo-4 images were used to generate a neuronal connectivity map of a relatively large field of view. Upon cessation of the stimulation the fluorescent signal recovered to control level.

The connectivity map (**Figure 7**) was generated by subtracting each image from the previous one. Eight consecutive subtracted frames, from the stimulation onset, were then sequentially color coded in accordance to a lookup color table. The total number of cell bodies activated by the stimulation was counted and the largest distance between the stimulated cell and the follower cells within the field of view was measured.

### SCANNING ELECTRON MICROSCOPY

For SEM analysis cells cultured on the NCD or glass substrate were fixed, dehydrated, within the culturing dish as previously described (Spira et al., 2003). Briefly, hippocampal primary cultured cells were fixed by 3% glutaraldehyde (Electron Microscopy Science) in cacodylate buffer (Agar Scientific, Stansted) at pH7.4. The cells were then washed in cold 0.1 M cacodylate buffer, pH7.4. The cells were post-fixed in 1% osmium tetroxide (Electron Microscopy Science) and 1.5% K_3_Fe(CN)_6_ (Sigma–Aldrich). Dehydration was carried out through a series of ethanol solutions and washed two times for 30 min with fresh 100% EtOH before critical point drying with liquid CO_2_ in a SAMDRI-PVT-3D (Tousimis, USA). Once dry the samples were sputtered with gold in an SPI-Module^TM^ Sputter Coater Module (SPI Supplies, USA). Images were taken with an Extra High Resolution Scanning Electron Microscopy MagellanTM 400L using an accelerating voltage of 5 kV.

### STATISTICAL ANALYSIS

The averaged density of cells adhering to PDL functionalized control glass or NCD substrates were calculated by averaging the densities (cells number/standard surface area of 607 × 607 μm) obtained from five randomly selected areas on days 3, 7, and 10 in culture (DIV). The data are presented as mean ± standard deviation. *t*-tests was performed using Excel software.

## RESULTS

### SYNTHESIS OF NCD FILMS

**FIGURE 1:**
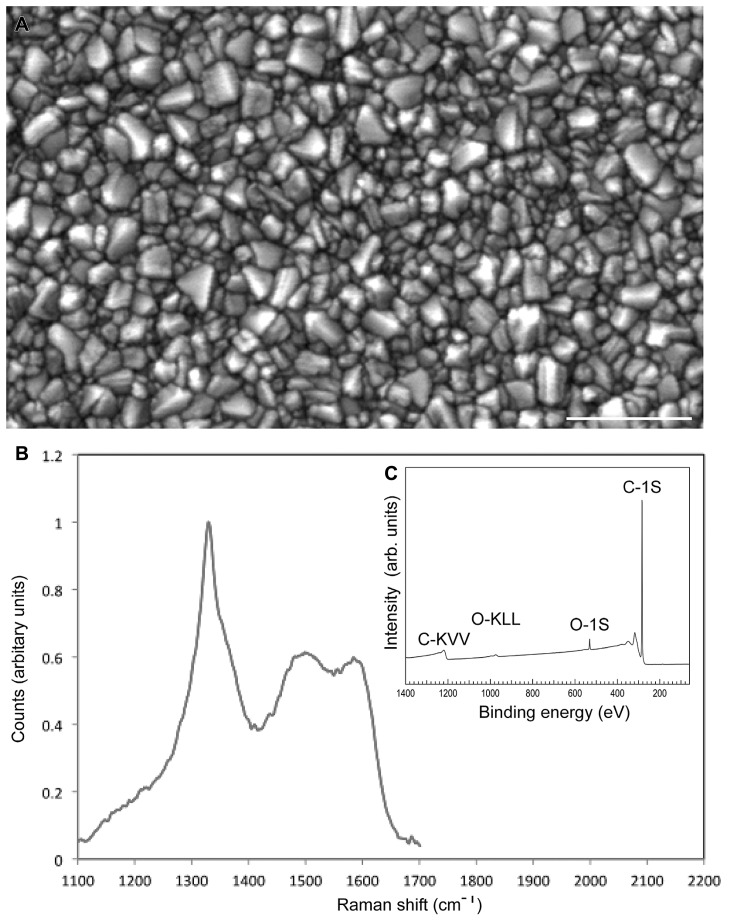
**Characterization of the nanocrystalline surface. (A)** SEM image of the nanocrystalline diamond (NCD) film morphology with Rmns ~17 nm. **(B)** The Raman spectra of the NCD film showing the zone-center phonon sp^3^ diamond line at 1332 cm^-1^ and a higher wave number signal, consisting of disordered and sp^2^ bonded formed at diamond gain boundaries. Content of non-diamond carbon is estimated to be 0.7%. **(C)** shows an XPS spectrum of oxidized NCD surface. Bar in A = 0.5 μm.

For the present study, 150 nm thick high quality NCD films were synthesized on fused silica substrate (10 mm × 5 mm × 0.5 mm size). Substrates were seeded in an aqueous suspension of DND particles to promote the diamond crystal nucleation and subsequently to initiate diamond layer growth (Barros et al., 2011). NCD films were grown in a resonance cavity microwave plasma enhanced chemical vapor deposition system (MW-PECVD) using 1% CH_4_ mixture in hydrogen at substrate temperature of 700°C (see Experimental methods for details: Williams et al., 2008; Daenen et al., 2009). The morphology of NCD films was composed of well-developed faceted nano-crystals of random orientation and is shown in **Figure 1A**. **Figure 1B** shows the Raman spectra of NCD films. The sp2 and disordered carbon originating at the grain boundaries, as typical from NCD films, was as low as 0.7% as determined by the numerical fitting of Raman spectra (for detail Ferrari and Robertson, 2004). The inset of **Figure 1C** shows an XPS survey scan of an oxidized NCD surface, acquired immediately after the MW-PECVD preparation and oxidizing the NCD diamond film in Ozone. Clearly, the spectrum displays ultra-clean diamond surface dominated only by the emission from carbon and oxygen. The C-1s core level (binding energy 284 eV) and in addition to C-lines, an O-1s signal are visible (531 eV) which can be assigned to the oxygen-terminated surface. Such surfaces were further used for neuron adhesion study.

### INCOMPATIBILITY OF NCD SUBSTRATE FOR PRIMARY NEURON ADHESION AND GROWTH

We tested the compatibility of bare NCD surface for adhesion and growth by culturing primary embryonic (E17) or newborn rat hippocampal cells on NCD, with hydrogen-(H-hydrophobic) and oxygen (O-hydrophilic)-termination. As a control to these experiments, we cultured neurons in the same experimental sessions on bare- and PDL-laminin functionalized glass substrates. The criteria used for comparing the cultures qualities were: adhesion of the cells to the substrate, the development of neurites on the substrate, survival time of the culture, and neuronal network functioning as revealed by calcium imaging of evoked network activity.

**FIGURE 2:**
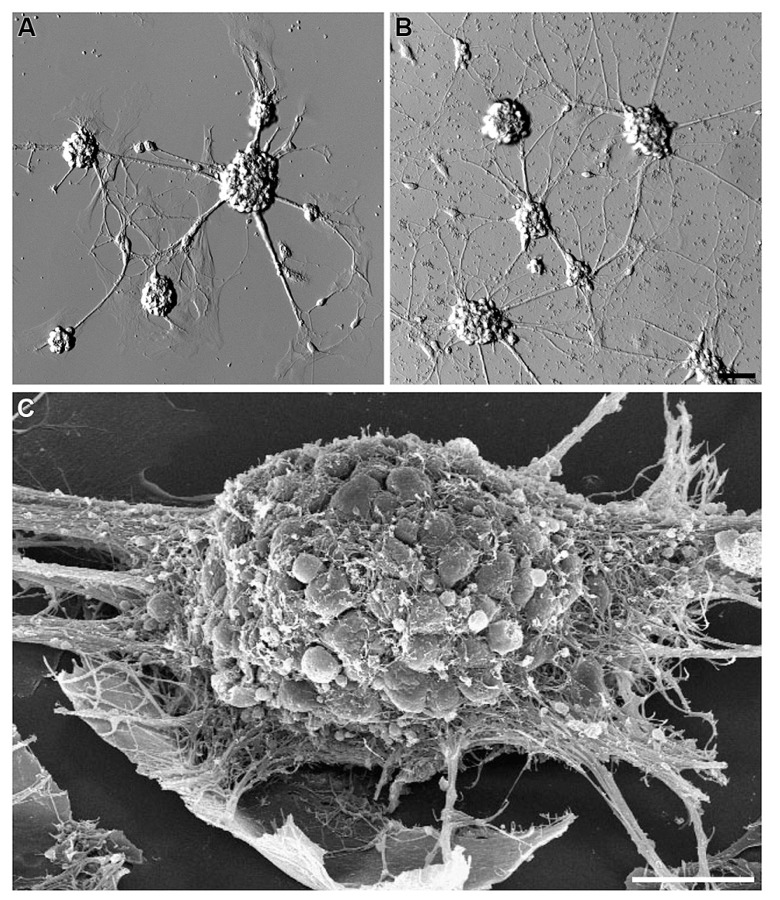
**Clusters of hippocampal neurons cultured on bare glass or NCD surfaces.** Embryonic (E17) hippocampal neurons cultured on bare glass **(A)** and bare NCD **(B)**, 3 DIV; bar = 50 μm.** (C)** SEM image of hippocampal neurons cultured on bare glass, 7 DIV; bar = 20 μm.

**FIGURE 3:**
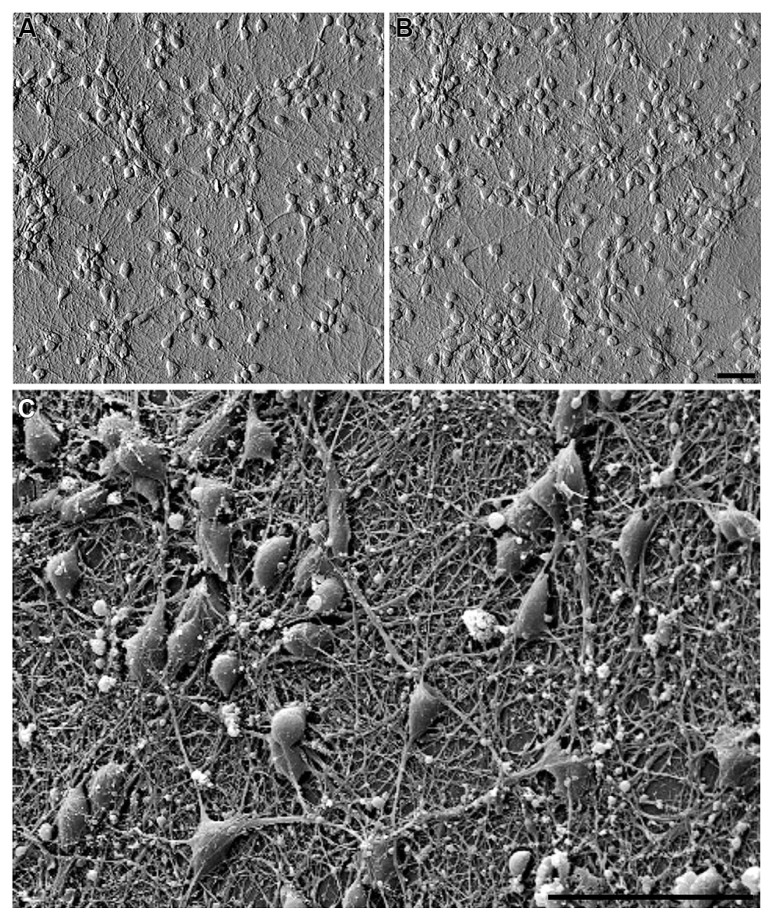
**Embryonic hippocampal neurons (7 DIV) cultured on: (A)** PDL-laminin functionalized glass and **(B)** PDL-laminin functionalized NCD. No difference in adhesion and growth is seen on the two substrates; bar = 50 μm. **(C)** SEM image of embryonic hippocampal neurons cultured on PDL-laminin functionalized NCD. Note the dense growth of neuritis; bar = 50 μm.

**FIGURE 4:**
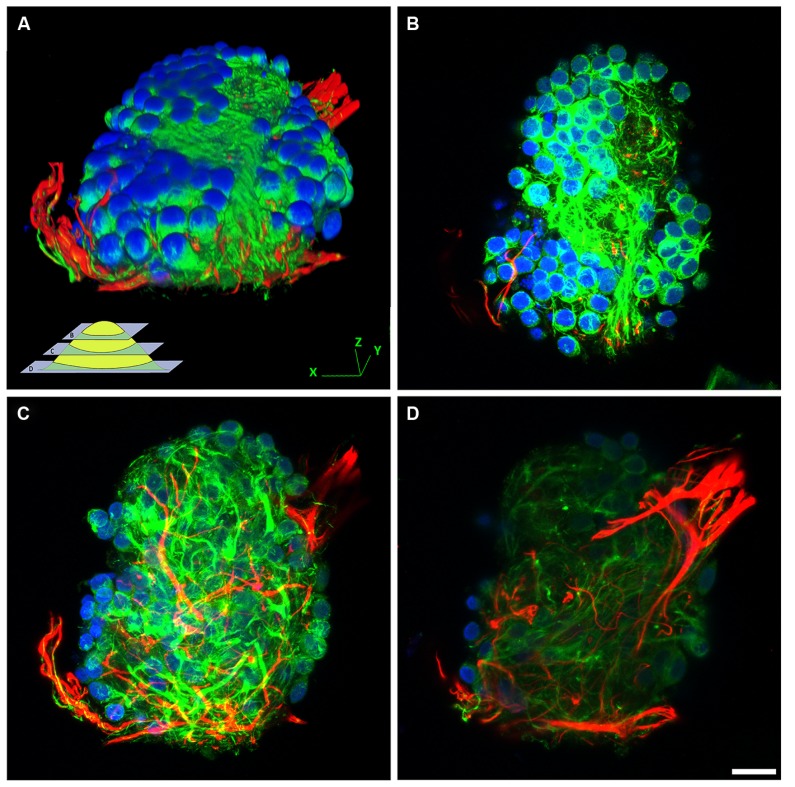
**The structural organization of cell–aggregates formed on bare NCD substrate.** Confocal image of immunolabeled (green- NF, red- GFAP, blue- DAPI) hippocampal neurons and glia cultured on bare NCD, 10 DIV. **(A)** Three dimensional reconstructed view of a cell-cluster. The insert illustrate the focal planes of the images shown in **B**, **C**, and **D**. **(B)** Top layer of the cell cluster (a focal slice of 18 μm), consists mainly of neuronal cell bodies. **(C)** Middle part of the cell-cluster (thickness of –19.5 μm), consists of neurites. **(D)** Bottom part of the cell cluster (slice thickness of –12.5 μm), close to the substrate level, showing glial processes; bar = 20 μm.

Hippocampal cells (neurons and glia) grown on bare NCD, or on bare glass substrates revealed poor adhesion and cell clustering (**Figure 2**). The cell clusters were interconnected by a small number of extensions. This is in contrast to control cultures of embryonic or new born hippocampal cells grown on PDL-Laminin functionalized glass-substrate which adhered well to the substrate and generated an elaborate network of neuritis (**Figure 3A**). The self-assembly of cells into clusters, rather than adhesion of individual cells to the substrate, reduces the contact surface area between the cells and the substrate, and enhances cell–cell contact. Such self-assembly of aggregates reflects significant adhesion-incompatibility between the cells and the substrate relative to the adhesion among cells. Three dimensional reconstructions of confocal microscope optical sections obtained at 0.25 μm steps revealed that in newborn cultures (that are rich with glia) the cells that interface with the substrate are mainly glia, whereas the neuronal cell bodies occupy the upper layers facing the bathing solution (**Figure 4**). Cultures prepared from embryos (E17) are almost glia-free, nevertheless they also form aggregates. These clusters detach from the substrate and degenerate within a number of days. No significant differences in cell-substrate adhesion were noticed among hippocampal cells cultured on bare glass substrate and the various NCD types used.

We conclude that post mitotic primary hippocampal neurons and glia grown on un-functionalized NCD or glass form cells aggregates as they do not adhere well to the substrate.

### HIPPOCAMPAL CELL ADHESION AND GROWTH ON FUNCTIONALIZED NCD SUBSTRATES

**FIGURE 5:**
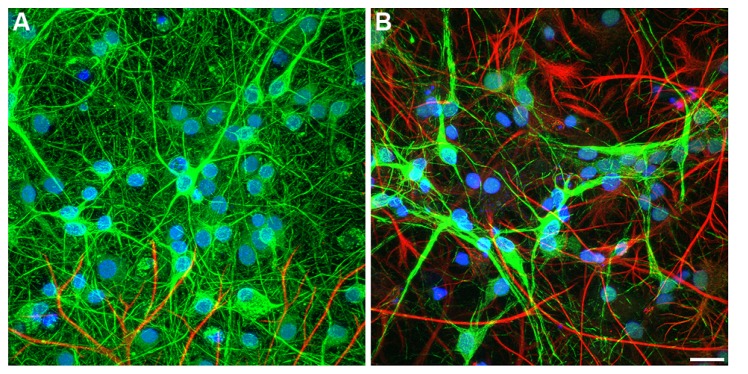
**Confocal image of immunolabeled hippocampal cells. (A)** embryonic, **(B)** newborn neurons cultured on PDL-laminin functionalized NCD, 10 DIV. The embryonic culture consists of mainly of neurons whereas newborns culture conssit of mixed neuron-glia cells. Both embryonic and new born cultures survive on functionalized NCD for over 3 weeks; bar = 20 μm.

**FIGURE 6:**
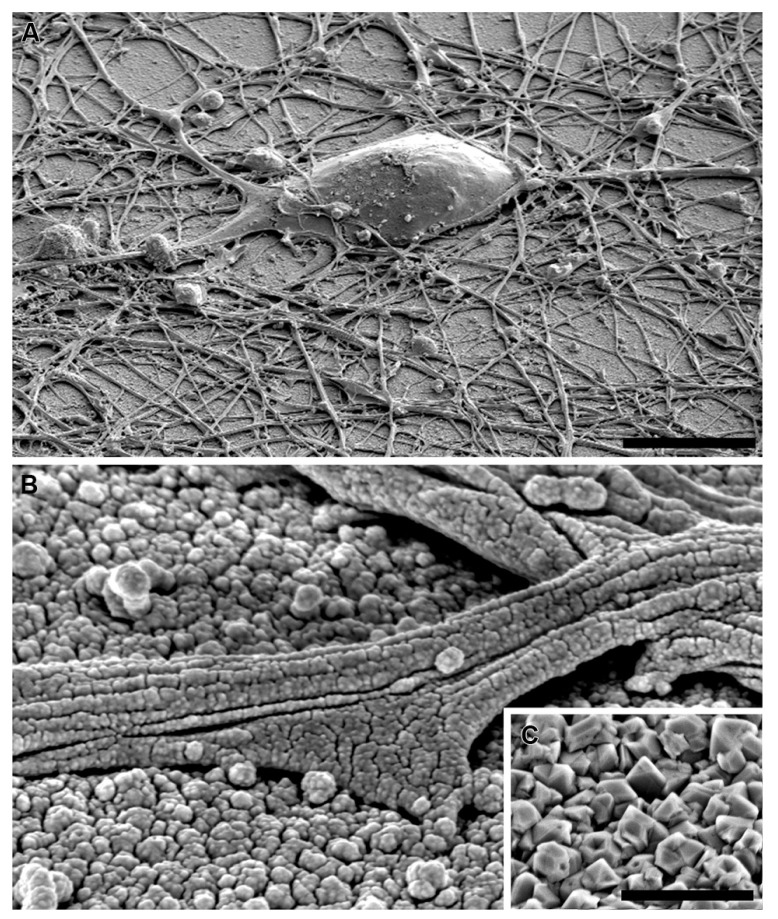
**SEM image of embryonic hippocampal neurons cultured on PDL-laminin functionalized NCD, 7 DIV. (A)** low magnification of a neuron; bar = 10 μm. **(B)** a neurite adhering to the ND layer. **(C)** NCD imaged without gold spattering; bar = 500 nm.

We next examined whether chemical functionalization of NCD surface solves the problem of cell-adhesion incompatibility to NCD substrates. To that end we employed the classical and robust PDL-laminin glass surface coating procedure. We found that functionalization of NCD substrates by PDL-laminin generates an adhesive and permissive substrate for primary neurons culturing (**Figures 3, 5** and **6**). Under these conditions, both embryonic and new born hippocampal cells adhere and extend a dense network of neuritis on the substrate. No significant differences in adhesion and survival over the time of observations were noted between neurons grown on functionalized glass or the various NCD substrates using morphological criteria (**Figure 3**) and functional assessments (see Neuronal Network Activity on Functionalized NCD Substrates). Thus, for cultures prepared from 17 days old embryos: on DIV 3 the averaged density of neurons on a standard area of functionalized glass (number of counted areas *n* = 5) was 683 ± 94 for control and on functionalized NCD 718 ± 173. These values are not significantly different (α = 0.05, *p* = 0.7). Likewise, the cell densities on DIV 7 and 10 did not change overtime on both the functionalized glass and NCD substrates exhibiting on DIV 7 densities of 528 ± 123 in control and 496 ± 151 on functionalized NCD (*p* = 0.7), and on DIV 10 527 ± 151 in control and 630 ± 137 on functionalized NCD (*p* = 0.29). The density of cultured cells (neurons and astrocytes) prepared from new born rats did not reveal any differences between the functionalized glass and NCD substrate but revealed a significant increase in the number of astrocytes on day 10 of the culture. Thus, on DIV 3 the averaged density of cells in the control was 247 ± 14 and on NCD 273 ± 105 (α = 0.05, *p* = 0.6), on DIV 7 in control the average was 177 ± 15 and on functionalized NCD 229 ± 46 (*p* = 0.01). On DIV 10 of the cultures prepared from new borne rats a large and significant increase in the density of the cells was observer on both glass and NCD substrates. The averaged density on glass was 437 ± 46 and on NCD 524 ± 153 (*p* = 0.26). The significant increase in the number of cells from DIV 7 to 10 is due to glia cell proliferation.

### NEURONAL NETWORK ACTIVITY ON FUNCTIONALIZED NCD SUBSTRATES

**FIGURE 7:**
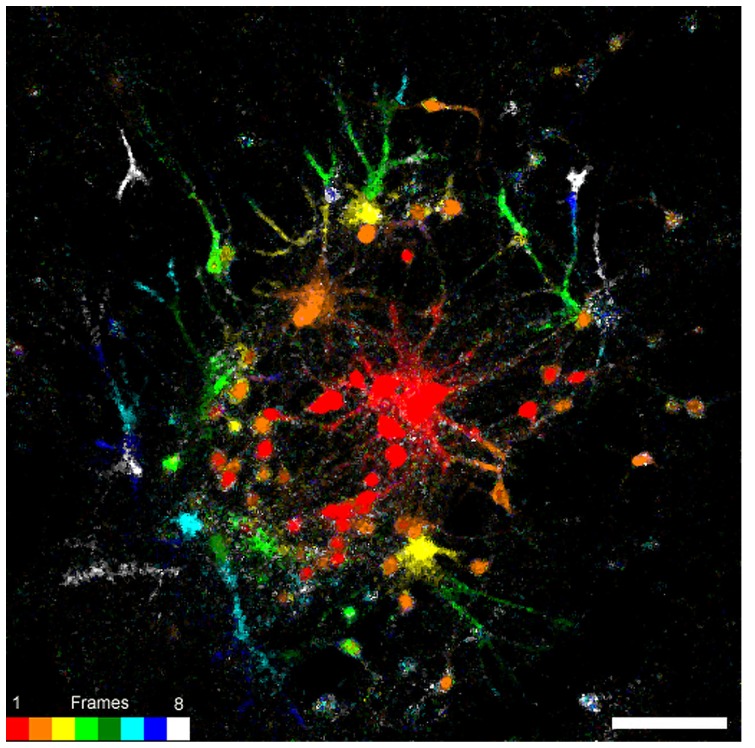
**Connectivity map of 14 days old cultured embryonic neurons on PDL-laminin functionalized NCD surface.** Nine consecutive confocal images were grabbed. The first before the onset of stimulation and the rest during and after the stimulation. Consequent images were subtracted (image n+1 - image n) to generate eight subtracted images. The resulting images were color coded accordance to the lookup *r* table attached to the figure. Note that the fluo-4 fluorescent signals propagated radially (from red to white along the pseudo color lookup table). The average number of cells that were excited by stimulation of a single neuron and the maximal distance of signal propagation was similar in cultures grown on functionalized glass and NCD. Bar = 20 μm.

The physical, chemical, and morphological nature of the substrates to which cells adhere and on which cells develop may have significant effects on cells morphology, their biophysical properties (electroanatomy) and, in the case of neurons, their synaptic functions and the overall network activities [for example (Fabbro et al., 2013)]. To assess whether neurons grown on NCD functionalized with PDL-Laminin maintain excitable membrane properties that promote propagation of action potentials along neurites, form functional connectivity that permit polysynaptic communication, we imaged the fluorescent signals generated by the calcium indicator fluo-4 in response to a long lasting train of stimuli delivered to a single neuron at the center of the field of view. To that end, the neurons were loaded with acetoxymethyl (AM) ester fluo-4 and then after removal of the excess fluo4-AM and incubation period to allow hydrolyze of the ester, a neuron located at the center of field was stimulated by a fire polished microelectrode. Live confocal imaging of the fluo-4 fluorescent revealed the propagation of the fluorescent signal from the stimulated cell to neighboring cell bodies along neurites (**Figure 7**). Analysis of the number of neurons activated by the stimulation in control (neurons cultured on functionalized glass substrate) and neurons grown on functionalized NCD were similar (45 ± 13.55 in control and 61.17 ± 41.04 on the NCD substrate. *t*-test for unequal variances *n* = 5 and 6, respectively, is for α = 0.05, *p* = 0.42). The maximal distance of the fluorescent signals propagation were the same ranging between 254 and 423 μm (322 ± 75 in control and 317 ± 66 on the NCD substrate. *t*-test for unequal variances *n* = 5 and 6, respectively, is for α = 0.005, *p* = 0.91). We conclude that the overall network connectivity of cells grown on functionalized NCD is similar to those grown on functionalized glass.

## DISCUSSION

The main observations of the present study is that in contrast to the general impression generated by earlier studies we found that post-mitotic primary rat hippocampal neurons and glial cells do not adhere and develop when cultured in defined medium on bare, oxidized and H-terminated NCD substrates. Nevertheless, classical chemical functionalization of the NCD substrate by PDL-laminin renders the substrate with permissive growth properties for these cells. Under these culturing conditions primary hippocampal neurons develop excitable membrane properties and excitatory synapses that communicate among them.

The incompatibility of bare NCD as a substrate for primary neuron culture reported here is in contradiction with earlier studies reporting that cell lines as well as hippocampal neurons adhere and develop on various NCD substrates without chemical surface functionalization prior to cell seeding. We devote the following paragraph to briefly propose a possible mechanism to account for this contradiction.

Under *in vivo* conditions cells are attached to each other by extracellular matrix (ECM) and cell adhesion molecules (CAMs; Abedin and King, 2010). Whereas the ECM and the CAM are secreted and expressed (respectively) by all cell types derived from multicellular organisms, their molecular, biochemical nature and levels of expression differ in different cell types and under different physiological conditions (for example see Barros et al., 2011). These differences are so substantial that *in vitro* cell culturing methods take advantage of the differences to isolate certain cell types from others. This is done, for example, by seeding a heterogeneous cell suspension on a common un-functionalized glass or plastic substrates. Under these conditions some cell types adhere to the bare substrate while others remain in suspension for a longer period of time (see for Danoviz and Yablonka-Reuveni, 2012). The selective cells-adhesivity reflects differences in the nature and rates of ECM secretion and CAM expression. The non-adhering or the adhering cells can then be collected. Differences in ECM molecular nature and secretion are also observed under *in vivo* conditions. For example regrowth of peripheral axons depend to a large extent on ECM secreted by supporting Schwann cells (Gonzalez-Perez et al., 2013). For these reasons, most classical and contemporary primary neuron culturing-procedures heavily rely on conscious chemical functionalization of the substrates prior to cells seeding.

It is conceivable that cell lines and primary proliferating cell types that are characterized by effective ECM secretion adhere and divide when plated on chemically un-functionalized surfaces. On the other hand, generations of cell biologists have established that *in vitro* culturing of primary neurons on glass substrate require surface functionalization prior to seeding. Therefore the reports of Thalhammer et al. (2010) and Edgington et al. (2013) who demonstrated that primary neurons which do not adhere to NCD adhere and grow on non-functionalized DND are somewhat surprising. The adhesion to DND could be explained by assuming that in these studies the DND surface was unconsciously functionalized by serum protein during the incubation step of the substrate in the seeding medium which contained 10% FBS. Interestingly, the protocol used by us also involved a ~20 h incubation period in 5% FBS. Nevertheless, as evident by our study, this and even extended periods of incubation, were insufficient to confer adhesive properties to our NCD substrate. The neurons did not adhere to sp3 carbon reach NCD grains neither to sp2 carbon rich grain boundaries. Both oxygen terminated (negative surface charge) or H-terminated bare NCD surfaces (positive surface charge) yielded to same negative result.

A possible mechanism that could account for this phenomenon could be related to differences in protein absorption capacities of DND and NCD films as the DND used by Thalhammer et al. (2010) and Edgington et al. (2013) are composed of round diamond particles of diameter ~8–10 nm while NCD films used in this study are composed of faceted crystals with Rms ~15–17 nm.

In conclusion, we demonstrate that bare NCD based substrates are not providing permissive adhesion and growth substrate for cultured primary neurons and glia. The literature demonstrates that cell lines and other proliferating cells can adhere and proliferate on ND substrates relying on their innate ECM resources. In contrast, cells that do not secret effective ECM (as primary neurons) will not adhere to NCD substrate and degenerate. We conclude that, whereas bare NCD has a broad range of advantageous material properties unusual cell adhesion properties are not included among them. Nevertheless, simple functionalization strategies make NCD a permissive substrate for adhesion and growth of post mitotic primary neurons. As functionalized NCD surfaces supports well neural adhesion its unique electrical mechanical and chemical properties is a highly attractive material for construction of *in vitro* and *in vivo* MEAs and BMIs.

## Conflict of Interest Statement

The authors declare that the research was conducted in the absence of any commercial or financial relationships that could be construed as a potential conflict of interest.
