# Corrigendum: Nanocrystalline diamond surfaces for adhesion and growth of primary neurons, conflicting results and rational explanation

**DOI:** 10.3389/fneng.2014.00037

**Published:** 2014-09-18

**Authors:** Matthew McDonald

**Affiliations:** Institute for Materials Research in MicroElectronics (IMO-MEC), Hasselt UniversityDiepenbeek, Belgium

**Keywords:** nanocrystalline diamonds, corrigendum, adhesion, growth, neurons

The article “Nanocrystalline diamond surfaces for adhesion and growth of primary neurons, conflicting results and rational explanation,” published 11 June 2014, with myself as the second author has a mispelling of my name. It came to my attention through a friend that my name is spelled incorrectly, which I did not notice during the review process.

On the paper, currently my name is spelled as: Mathew McDonald. The correct spelling is: Matthew McDonald.

## Conflict of interest statement

The author declares that the research was conducted in the absence of any commercial or financial relationships that could be construed as a potential conflict of interest.

